# Necrotizing Enterocolitis in Term Neonates with Critical Congenital Heart Disease: Associated Risk Factors and Clinical Outcomes

**DOI:** 10.3390/jcdd13070329

**Published:** 2026-07-14

**Authors:** Demet Kangel, Eymen Recep, Burcu Çevlik, Ahmet Saki Oğuz, Berra Zümrüt Tan Recep, Halise Zeynep Genç, Dilek Yavuzcan Öztürk, Ali Can Hatemi, Erkut Öztürk

**Affiliations:** 1 Department of Pediatric Cardiology, Saglik Bilimleri University Basaksehir Cam and Sakura City Hospital, Istanbul 34480, Turkey; drburcudrl@gmail.com (B.Ç.); ahmetsaki@gmail.com (A.S.O.); zeynep.iscan@hotmail.com (H.Z.G.); erkut.ozturk@sbu.edu.tr (E.Ö.); 2 Department of Pediatric Cardiovascular Surgery, Saglik Bilimleri University Basaksehir Cam and Sakura City Hospital, Istanbul 34480, Turkey; dreymenrecep@gmail.com (E.R.); berrazumrut.tanrecep@sbu.edu.tr (B.Z.T.R.); alican.hatemi@sbu.edu.tr (A.C.H.); 3 Department of Neonatology, Saglik Bilimleri University Basaksehir Cam and Sakura City Hospital, Istanbul 34480, Turkey

**Keywords:** congenital heart disease, necrotizing enterocolitis, term neonate, critical CHD, risk factors

## Abstract

**Background:** Necrotizing enterocolitis (NEC) is a severe gastrointestinal complication that may develop in term neonates with critical congenital heart disease (CHD), despite the absence of prematurity. We aimed to evaluate the incidence of NEC and identify clinical factors associated with NEC development in term neonates with critical CHD. **Methods:** This retrospective cohort study included term neonates (≥37 weeks’ gestation) with critical CHD admitted to a tertiary pediatric cardiac intensive care unit between 1 January 2022, and 1 January 2026. NEC was defined as stage IIA or higher according to modified Bell’s criteria. Demographic, clinical, perioperative, and nutritional variables were analyzed. Multivariable logistic regression analysis was performed to identify factors independently associated with NEC development. **Results:** A total of 780 term neonates with critical CHD were included. NEC developed in 18 infants (2.3%). The median age at NEC diagnosis was 7 days (IQR 5–10), and four patients (22%) required surgical intervention. Seven NEC cases occurred preoperatively, whereas 11 developed after cardiac surgery; among postoperative cases, 64% occurred within the first 72 h after surgery. Birth weight < 2.5 kg (aOR 4.1, 95% CI 1.2–13.4; *p* = 0.021), gestational age < 38 weeks (aOR 3.9, 95% CI 1.3–12.0; *p* = 0.018), functional single-ventricle physiology (aOR 5.8, 95% CI 1.6–20.7; *p* = 0.007), and mechanical ventilation dependency (aOR 5.1, 95% CI 1.7–15.2; *p* = 0.004) were independently associated with NEC development. Mortality was significantly higher among infants with NEC compared with those without NEC (50% vs. 7.7%, *p* < 0.001). **Conclusions:** NEC remains an important complication associated with substantial mortality in term neonates with critical CHD, despite its relatively low incidence. Early-term birth, low birth weight, functional single-ventricle physiology, and dependence on mechanical ventilation were independently associated with increased NEC risk in this vulnerable population.

## 1. Introduction

Congenital heart disease (CHD) is among the most common congenital anomalies in newborns. Previous epidemiological studies have reported that the incidence of moderate-to-severe CHD is approximately 6 per 1000 live births [1]. CHD remains one of the leading causes of neonatal morbidity and mortality requiring intensive care, catheter-based intervention, or surgical treatment during early infancy [2,3]. Despite significant advances in perioperative management and neonatal cardiac surgery, these infants remain particularly vulnerable to systemic hypoperfusion, end-organ dysfunction, and perioperative complications related to impaired tissue oxygen delivery [2,4].

Necrotizing enterocolitis (NEC) is a severe inflammatory intestinal disorder characterized by mucosal injury, ischemia, bacterial translocation, and varying degrees of bowel necrosis [5,6]. Although classically associated with prematurity, NEC may also develop in term neonates with critical CHD through distinct pathophysiological mechanisms, including hemodynamic instability, chronic systemic hypoxemia, impaired mesenteric perfusion, low diastolic pressure in duct-dependent lesions, abdominal aortic runoff, and perioperative low cardiac output states, rather than the immaturity-driven pathways characteristic of preterm NEC [3,5,6,7]. Prolonged mechanical ventilation, delayed enteral feeding, and dependence on parenteral nutrition may further compromise intestinal integrity in this population [3,8,9]. In infants with CHD, NEC is associated with prolonged hospitalization, increased need for surgical intervention, and substantially higher mortality rates [6,8,10].

Previous studies evaluating NEC in neonates with CHD have reported highly variable incidence rates and heterogeneous risk factors [3,5,7,11]. Recent literature has also emphasized the lack of standardized diagnostic approaches for CHD-associated NEC, which may further contribute to variability in reported incidence rates and clinical characteristics among studies [12]. However, many of these studies included both preterm and term infants or heterogeneous cardiac populations, limiting interpretation of NEC risk specifically in term neonates with critical CHD [6,13]. Data specifically focusing on term infants with critical CHD remain limited, particularly in contemporary large-volume pediatric cardiac centers [3,8].

An earlier analysis from our institution examined NEC in a smaller cohort of term neonates with critical CHD over a shorter observation period and identified single-ventricle physiology and parenteral nutrition exposure as potential risk factors; however, that study was limited by its sample size and the absence of multivariable adjustment [8]. The present study substantially expands the patient population (n = 780 vs. the prior cohort), extends the study period to four years, and applies multivariable logistic regression to identify independent predictors of NEC development in this vulnerable group.

The aim of this study was to evaluate the incidence of NEC and identify clinical and hemodynamic factors associated with NEC development in term neonates with critical CHD admitted to a tertiary pediatric cardiac intensive care unit.

## 2. Methods

### 2.1. Study Population and Design

This retrospective cohort study was conducted in a tertiary pediatric cardiac intensive care unit and included term neonates with critical CHD who were hospitalized between 1 January 2022 and 1 January 2026.

To minimize the confounding effects of prematurity on NEC development, only neonates born at ≥37 weeks of gestation were included in the analysis. Infants with congenital gastrointestinal anomalies such as gastroschisis and duodenal atresia were excluded because these conditions may independently increase the risk of intestinal morbidity. In addition, cases of isolated postoperative pneumoperitoneum and infants diagnosed with NEC beyond 90 days of life were not included in the study population.

The study protocol was approved by the local ethics committee (Approval No: 2026-226; Approval Date: 29 April 2026), and all study procedures were conducted in accordance with the principles outlined in the Declaration of Helsinki.

Full-term birth was defined as a gestational age of at least 37 completed weeks. Critical CHD was classified into four hemodynamic groups: (i) duct-dependent pulmonary circulation lesions, including pulmonary atresia and tetralogy of Fallot; (ii) duct-dependent systemic circulation lesions, including hypoplastic left heart syndrome, coarctation of the aorta, and interrupted aortic arch; (iii) lesions associated with inadequate intercirculatory mixing, such as transposition of the great arteries; and (iv) other complex congenital cardiac anomalies, including truncus arteriosus and total anomalous pulmonary venous return [2].

### 2.2. Data Collection and Definitions

Demographic and clinical characteristics were obtained from electronic medical records. Collected variables included gestational age, birth weight, cardiac diagnosis, feeding status at the time of NEC diagnosis, requirement for mechanical ventilation, age at NEC onset, and type of NEC treatment (medical or surgical).

Low cardiac output syndrome (LCOS) was defined clinically based on signs of inadequate systemic perfusion requiring escalation of vasoactive support, including prolonged capillary refill, oliguria, metabolic acidosis, or elevated lactate levels.

Operative variables included cardiopulmonary bypass duration, aortic cross-clamp time, and surgical complexity based on the Risk Adjustment for Congenital Heart Surgery-1 (RACHS-1) classification. RACHS-1 categories were assigned according to previously published criteria [14]. In our institution, the red blood cell transfusion protocol consisted of administering 15 mL/kg leukoreduced and irradiated packed red blood cells. Target hematocrit values were generally maintained at ≥40% in neonates with cyanotic cardiac lesions and ≥35% in those with non-cyanotic lesions.

Enteral feeding practices in our unit were individualized according to the patient’s hemodynamic status and clinical stability. Trophic feeding and feed advancement decisions were made by the multidisciplinary intensive care team based on perfusion status, vasoactive support requirement, abdominal examination findings, and perioperative clinical condition. In clinically unstable patients or during perioperative fasting periods, parenteral nutrition was preferred until enteral feeding was considered safe.

The diagnosis of NEC was established according to modified Bell’s criteria. Only patients with stage IIA or higher disease were included in the NEC group [15,16]. NEC staging was determined using both clinical and radiologic findings, including feeding intolerance, abdominal distention, apnea, pneumatosis intestinalis, portal venous gas, and pneumoperitoneum. Following the diagnosis of NEC, all patients received bowel rest, gastric decompression, parenteral nutrition, and empirical intravenous ampicillin/sulbactam plus cefotaxime. Antibiotic therapy was continued for 7–14 days according to Bell stage and clinical response and was modified when culture results became available. Patients undergoing surgery received perioperative cefazolin prophylaxis. In patients with positive blood cultures, antimicrobial therapy was tailored according to the identified pathogen and antimicrobial susceptibility testing in consultation with the pediatric infectious diseases team.

The primary outcome of the study was the development of NEC in term neonates with critical CHD during hospitalization. Secondary outcomes included mortality, requirement for surgical treatment of NEC, duration of intensive care unit stay, duration of hospitalization, and identification of clinical factors associated with NEC development.

### 2.3. Statistical Analysis

Statistical analyses were performed using IBM SPSS Statistics for Windows, version 21.0 (IBM Corp., Armonk, NY, USA). Normality of continuous variables was assessed using the Shapiro–Wilk test. Because most continuous variables demonstrated non-normal distribution, they were expressed as median and interquartile range (IQR). Categorical variables were expressed as numbers and percentages. Group comparisons between NEC and non-NEC patients were performed using the Mann–Whitney U test for continuous variables and the chi-square or Fisher’s exact test for categorical variables, as appropriate. Because early-term delivery may be associated with adverse neonatal outcomes, the relationship between gestational age and NEC development was additionally evaluated by comparing infants born at <38 weeks and ≥38 weeks of gestation. Low birth weight was defined as a birth weight below 2500 g, and its association with NEC development was also assessed based on previous reports in infants with CHD [3,10]. Variables demonstrating *p* values < 0.10 in univariate analyses were considered for inclusion in the multivariable logistic regression model. Final variable selection was additionally guided by clinical relevance, potential collinearity among hemodynamic variables, and the events-per-variable principle in order to reduce model overfitting and improve model stability. Given the limited number of NEC events (n = 18), variables considered to represent outcomes of NEC or that occur concurrently with or after its development—including low cardiac output syndrome, length of ICU and hospital stay, mortality, and transfusion-related variables—were excluded a priori from the multivariable model to avoid reverse-causation bias. Among the remaining preoperative and intraoperative candidate variables, birth weight < 2.5 kg, gestational age, functional single-ventricle physiology, and mechanical ventilation dependency were retained in the final model based on biological plausibility and low mutual collinearity. Adjusted odds ratios (aORs) and corresponding 95% confidence intervals (CIs) were calculated. A two-sided *p*-value < 0.05 was considered statistically significant.

## 3. Results

A total of 780 term neonates with critical CHD were included during the study period, including 406 males (52%). Baseline demographic and clinical characteristics of the study population are presented in Table 1. The median gestational age was 39.0 weeks (IQR 38–40), and the median birth weight was 2900 g (IQR 2800–3000).

Necrotizing enterocolitis developed in 18 infants, corresponding to an incidence of 2.3%. Sixteen NEC cases occurred in neonates with duct-dependent cardiac lesions (16/624), whereas two cases were observed among infants with other forms of critical CHD (2/156). The median age at NEC diagnosis was 7 days (IQR 5–10). According to modified Bell staging criteria, 11 infants were classified as stage IIA, 5 as stage IIB, 1 as stage IIIA, and 1 as stage IIIB. Among the NEC cases, 7 developed during the preoperative period, whereas 11 occurred postoperatively. Of the postoperative NEC cases, 7 (64%) developed within the first 72 h after surgery and 4 (36%) occurred after 72 h. Four patients (22%) required surgical intervention (Table 2). All underwent laparotomy; three unstable infants with focal intestinal involvement were treated with stoma formation, whereas one infant with multifocal disease underwent a clip-and-drop procedure.

At the time of NEC diagnosis, 11 infants (61%) were receiving full enteral feeding, 2 (11%) were receiving combined enteral and parenteral nutrition, and 5 (28%) were exclusively dependent on total parenteral nutrition. Within the NEC cohort, median gestational age was 37.5 weeks (IQR 37–38), median birth weight was 2800 g (IQR 2600–3000), and median postnatal age at diagnosis was 7 days (IQR 5–10).

Mechanical ventilation support was required in 11 of 18 infants (61%) in the NEC group, compared with 114 of 762 infants (15%) in the non-NEC group. Several demographic and clinical variables differed significantly between groups, as summarized in Table 1. Among the 11 ventilated infants in the NEC group, the indication for mechanical ventilation was hemodynamic instability/low cardiac output in 6 (55%), primary pulmonary disease in 3 (27%), and airway abnormality in 2 (18%). A detailed indication-level classification was not systematically performed for ventilated infants in the non-NEC group, as ventilation status was recorded as a binary variable (present/absent) rather than by underlying indication.

Catheter-related sepsis occurred in 6 infants with NEC. Identified pathogens included Klebsiella pneumoniae (n = 3), Enterococcus faecalis (n = 1), Staphylococcus epidermidis (n = 1), and Candida albicans (n = 1). Median CRP at NEC diagnosis was 28 mg/L (IQR 20–54). All infants received empirical ampicillin/sulbactam plus cefotaxime following NEC diagnosis; therapy was escalated to broader-spectrum or targeted antimicrobial regimens in culture-positive patients and in one culture-negative patient with clinical deterioration, with total treatment duration ranging from 7 to 14 days (Table 3). Among the 6 infants with catheter-related sepsis, the implicated vascular access was an umbilical arterial catheter in 3, a femoral venous catheter in 2, and a peripheral intravenous line in 1. Among the remaining 12 infants without catheter-related sepsis, vascular access included umbilical catheters in 4, femoral venous catheters in 3, jugular venous catheters in 3, and peripheral intravenous access only in 2.

Circulatory arrest was used in 4 of 18 infants (22%) in the NEC group compared with 63 of 762 infants (8.3%) in the non-NEC group; this difference did not reach statistical significance (*p* = 0.080).

In multivariable logistic regression analysis, birth weight < 2.5 kg, gestational age < 38 weeks, mechanical ventilation dependency, and functional single-ventricle physiology remained independently associated with NEC development (Table 4). Low birth weight was associated with an approximately fourfold increased risk of NEC (aOR 4.1, 95% CI 1.2–13.4; *p* = 0.021). Early-term birth was similarly associated with an approximately fourfold increased risk (aOR 3.9, 95% CI 1.3–12.0; *p* = 0.018). Mechanical ventilation dependency increased NEC risk more than fivefold (aOR 5.1, 95% CI 1.7–15.2; *p* = 0.004), and functional single-ventricle physiology was associated with an almost sixfold increased risk (aOR 5.8, 95% CI 1.6–20.7; *p* = 0.007). Model calibration demonstrated acceptable fit according to the Hosmer–Lemeshow test (*p* = 0.61). Transfusion burden and parenteral nutrition exposure were not independently associated with NEC after multivariable adjustment.

Mortality was significantly higher among infants who developed NEC. The case-fatality rate was 50% in the NEC group compared with 7.7% among infants without NEC (*p* < 0.001). The causes of death among the 9 infants who died included low cardiac output syndrome (n = 3), septicemia (n = 3), arrhythmia (n = 1), pulmonary hemorrhage (n = 1), and acute kidney injury (n = 1).

## 4. Discussion

In this study, we evaluated the incidence of NEC and associated clinical risk factors in term neonates with critical CHD managed in a tertiary pediatric cardiac intensive care unit. NEC developed in 2.3% of the study population, which is generally consistent with previously reported incidence rates among neonates with complex CHD, despite differences in patient selection and cardiac lesion profiles across studies [5,6,10,17]. We found that lower gestational age, functional single-ventricle physiology, and dependence on mechanical ventilation were independently associated with NEC development.

Previous studies have suggested that impaired systemic perfusion, chronic hypoxemia, and circulatory instability may contribute to intestinal vulnerability in infants with critical CHD [3,6,18,19,20,21]. Our findings are in line with these observations, particularly given the higher frequency of single-ventricle physiology, ventilator dependency, and low cardiac output syndrome among infants who developed NEC. In addition, the majority of postoperative NEC cases in our cohort occurred within the first 72 h after surgery, supporting the potential contribution of perioperative hemodynamic instability in this high-risk population. Given the limited number of studies focusing exclusively on term neonates with critical CHD, the present study provides additional contemporary data regarding clinical factors associated with NEC development in this vulnerable group.

In the general term neonatal population, NEC is relatively uncommon, with reported incidence rates ranging between 0.05 and 0.71 per 1000 live births [22,23]. However, neonates with critical CHD appear to have a substantially increased risk of NEC compared with otherwise healthy term infants. Previous studies have reported NEC incidences ranging from approximately 2.9% to 3.7% in infants with critical CHD, with even higher rates observed in duct-dependent lesions and more complex cardiac physiologies [5,10,18,24]. In a large retrospective cohort, Gong et al. reported NEC in approximately 16% of neonates with CHD, particularly among patients with duct-dependent circulation [7]. In our cohort, the overall NEC incidence was 2.3%, which is generally consistent with the lower range of previously published studies. Differences among studies may reflect variability in patient selection, cardiac lesion severity, perioperative management strategies, and institutional feeding practices.

One of the notable findings of our study was the association between NEC development and gestational age below 38 weeks, despite all infants being classified as term neonates. This observation suggests that early-term infants with critical CHD may represent a particularly vulnerable subgroup. Previous studies have shown that infants with complex CHD are exposed to multiple hemodynamic stressors, including systemic hypoxemia, reduced diastolic perfusion pressure, abdominal aortic runoff in duct-dependent lesions, and perioperative low cardiac output states, all of which may adversely affect mesenteric oxygen delivery [10,11,18,25,26]. In this setting, even modest differences in gestational maturity may contribute to reduced intestinal tolerance to circulatory instability and hypoperfusion.

Low birth weight (<2.5 kg) was also independently associated with NEC development in the present cohort. Although all infants were born at term, birth weight may serve as an additional marker of relative fetal growth restriction or subclinical hemodynamic compromise in the setting of critical CHD, potentially reflecting reduced placental or systemic perfusion in utero. This finding is consistent with previous reports linking lower birth weight to adverse gastrointestinal outcomes even among term infants with CHD [3], and suggests that birth weight may provide additional risk stratification value beyond gestational age alone.

Functional single-ventricle physiology was independently associated with NEC development in our cohort. Infants with single-ventricle circulation are particularly susceptible to circulatory instability because of parallel circulation physiology, limited systemic oxygen delivery, and reduced cardiac reserve, which may adversely affect mesenteric perfusion [4,8,19,27]. Similar findings have been reported by Choi et al., who identified single-ventricle physiology, lower gestational age, mechanical ventilation, and parenteral nutrition exposure as factors associated with NEC in term infants with critical CHD [3]. Together, these observations suggest that hemodynamic vulnerability may play an important role in NEC development in this patient population, in addition to gestational maturity alone.

Prostaglandin E1 exposure was common in both NEC and non-NEC groups and was not independently associated with NEC development in our cohort. Previous studies have reported conflicting findings regarding the relationship between prostaglandin therapy and NEC risk in neonates with duct-dependent CHD, partly because prostaglandin exposure may also reflect underlying lesion severity and circulatory instability rather than a direct treatment-related effect [6,17,28]. In our institution, low-dose prostaglandin protocols are routinely preferred whenever clinically feasible, which may have influenced the absence of a significant association in the present study.

The relationship between red blood cell transfusion and NEC remains controversial. Several mechanisms have been proposed, including alterations in mesenteric oxygen delivery, inflammatory responses, and impaired intestinal perfusion following transfusion exposure [16,25,29]. Baxi et al. reported an association between increased erythrocyte transfusion exposure and NEC development in neonates with CHD [24]. In our cohort, however, transfusion burden was not independently associated with NEC after multivariable adjustment. This finding may suggest that the need for transfusion reflects an underlying state of relative anemia and reduced systemic oxygen delivery—and, by extension, impaired mesenteric perfusion—rather than a direct causal effect of the transfusion itself. In this view, transfusion requirement may be better understood as a marker of gut ischemia risk than as an independent mechanistic trigger for NEC [30,31]. Further prospective studies are needed to better clarify this relationship.

The relationship between enteral feeding and NEC in infants with CHD remains complex and is likely influenced by both nutritional and hemodynamic factors. Iannucci et al. reported that nearly 60% of postoperative NEC cases developed after initiation of enteral feeding, although no significant differences in feeding characteristics were identified between NEC and non-NEC groups [13]. In contrast, Del Castillo et al. demonstrated that implementation of a structured and gradual postoperative feeding advancement protocol in infants with hypoplastic left heart syndrome was associated with reduced NEC incidence [11]. Furthermore, Cognata et al. showed that exclusive human milk feeding was associated with lower rates of preoperative NEC in neonates with complex CHD [9]. Human milk may provide protection through immunologic, anti-inflammatory, and microbiome-modulating mechanisms.

In our cohort, infants who developed NEC were more frequently exposed to partial or total parenteral nutrition, whereas human milk utilization remained relatively low. However, these findings should be interpreted cautiously, since nutritional management in critically ill neonates is strongly influenced by underlying hemodynamic instability and overall disease severity. Although parenteral nutrition may theoretically reduce intestinal stress during periods of circulatory compromise, prolonged dependence may also increase central venous catheter exposure and infection risk. Therefore, parenteral nutrition exposure may represent both a supportive therapeutic strategy and a marker of severe clinical status. Overall, these findings support the importance of individualized feeding approaches guided by hemodynamic stability rather than uniform feeding restriction or advancement protocols. Recent studies have similarly emphasized hemodynamic-focused and risk-stratified nutritional strategies in infants with CHD, highlighting the importance of tailoring feeding advancement according to circulatory status and lesion severity rather than adopting a uniform feeding approach [27,32,33].

Catheter-related sepsis was observed in a subset of infants who developed NEC and may have contributed to intestinal injury through systemic inflammation, impaired perfusion, and greater overall hemodynamic instability. In critically ill neonates with CHD, prolonged central venous access, parenteral nutrition exposure, and intensive care support may increase susceptibility to bloodstream infections, which can further compromise mesenteric circulation and intestinal integrity [27,28,34]. However, because of the retrospective design of the present study, the temporal relationship between sepsis and NEC development could not be definitively established.

Ischemia–reperfusion injury represents an additional mechanistic pathway that may contribute to intestinal mucosal injury in this population, particularly following circulatory arrest [35,36]. Circulatory arrest was used numerically more often in the NEC group (4/18, 22%) than in the non-NEC group (63/762, 8.3%), although this difference did not reach statistical significance (*p* = 0.080), likely reflecting the limited number of NEC events. This trend nonetheless raises the possibility that reperfusion-related systemic inflammation, in addition to sustained hypoperfusion, may have contributed to intestinal injury in a subset of postoperative NEC cases. Because inflammatory markers were not systematically available for the full cohort, this mechanism could not be directly tested and is acknowledged as a limitation.

Mortality among infants who developed NEC in our cohort reached 50%, substantially higher than in infants without NEC (7.7%). Similar findings have been reported in previous studies of neonates with critical CHD. Choi et al. demonstrated markedly higher mortality rates among term infants with CHD who developed NEC compared with those without NEC, while Moschino et al. also emphasized the severe clinical course and high mortality associated with CHD-related NEC [3,18]. Becker et al. similarly reported mortality rates approaching 47% among infants with duct-dependent CHD complicated by NEC [19]. Together, these findings suggest that NEC in the setting of critical CHD is strongly associated with severe hemodynamic compromise and adverse clinical outcomes.

### Limitations

This study has several limitations. First, because of its retrospective single-center design, the findings may not be fully generalizable to other populations. Second, although the overall cohort size was relatively large, the number of NEC events was limited, which may have affected the stability of the multivariable analysis. In addition, because of the limited number of NEC events (n = 18), the multivariable model could only accommodate a small number of variables, and several statistically significant univariate predictors could not be simultaneously included without increasing the risk of overfitting. Variable selection was therefore guided by clinical and biological relevance rather than statistical significance alone, and residual confounding from variables not included in the final model cannot be excluded. Some clinically relevant variables related to perioperative nutritional management and hemodynamic status could not be evaluated in detail for the same reason. In particular, although the indication for mechanical ventilation could be classified for NEC infants (hemodynamic instability in 55%, primary pulmonary disease in 27%, and airway abnormality in 18%), a comparable indication-level classification was not systematically performed for the non-NEC group, in which ventilation status was recorded only as present or absent, precluding a formal between-group comparison of ventilation indication. Furthermore, the high overall rate of prostaglandin E1 use and institutional preference for low-dose administration may have limited the ability to detect a possible association between PGE1 exposure and NEC development.

In conclusion, NEC remains a serious and clinically important complication in term infants with critical CHD, despite its relatively low incidence (2.3%). Lower gestational age, low birth weight, functional single-ventricle physiology, and dependence on mechanical ventilation were associated with an increased risk of NEC development in this vulnerable population.

## Figures and Tables

**Table 1:** Demographics and patient characteristics.

Variables	NEC (n = 18)	No NEC (n = 762)	*p*
Weight (kg)	2.8 [2.6–3.0]	3.0 [2.8–3.2]	0.02
Birth weight, kg ≥2.5 <2.5	14 (77) 4 (25)	716 (94) 46 (6)	**0.012**
Gestational Age/week	37.5 [37–38]	39 [38–40]	**0.035**
Mode of Delivery, cesarian section	9 (50)	344 (45)	0.760
Male	9 (50)	397 (52.1)	0.890
Saturation	87 [85–91]	89 [86–92]	0.840
Physiology Single ventricle Biventricular	12 (66) 6 (33)	214 (28) 548 (72)	**0.002**
Duct dependent pulmonary circulation Duct dependent systemic circulation Mixing Other	9 (50) 6 (33) 2 (11) 1 (5)	283 (37) 326 (43) 96 (12) 57 (8)	0.815
PGE1 use	16 (88)	647 (85)	0.890
Feeding type breast milk Formula milk Mixed type NPO with TPN	2 (11) 6 (33) 3 (17) 7 (38)	187 (24) 246 (32) 284 (37) 45 (6)	0.001
Feeding status Full enteral feeding Parenteral nutrition or NPO	11 (61) 7 (38)	717 (94) 45 (6)	**<0.001**
Ventilator care Yes No	11 (61) 7 (38)	114 (15) 648 (85)	**0.005**
Intervention Operation Catheter	15 (83) 3 (17)	666 (88) 96 (12)	0.556
RACHS-1 ≥ 4	14 (77)	511 (67)	0.120
Number of packed RBC transfusions < 6	3 (17)	717 (94)	**<0.001**
Median volume of RBCs transfused (mL)	95 [70–120]	60 [40–90]	0.440
ECMO	2 (11)	37 (5)	0.340
Vasoactive Inotrope Score < 10	12 (66)	572 (75)	0.080
Arrhythmias	2 (11)	61 (8)	0.891
Acute Kidney Injury	6 (33)	153 (20)	0.120
LCOS	14 (77)	228 (30)	**0.01**
ICU stay (days)	16 [14–18]	10 [7–15]	**0.015**
Post-op hospital stay (days)	30 [25–35]	18 [15–22]	**0.008**
Mortality	9 (50)	59 (7.7)	**<0.001**

Median (IQR) n (%) ECMO: Extracorporeal membrane oxygenation, ICU: Intensive care unit, LCOS: Low cardiac output syndrome, NEC: Necrotizing enterocolitis, NPO: nil per os, RACHS-1: Risk adjustment for congenital heart surgery, ref: references, RBCs: Red blood cells.

**Table 2:** Patient and clinical information of NEC.

Patient	Sex	Birth Weight/kg	Gestational Age/Week	Type of CHD	Age at NEC	Preop NEC	Feeding Status at NEC Diagnosis	NEC Bell’s Stage	Surgical Treatment of NEC	Mortality
1	Male	2.8	38	PA/IVS	6	No	Parenteral	IIa	No	Yes
2	Male	2.6	38	DILV	7	No	Enteral	IIb	No	No
3	Female	3	40	RAI	8	No	Enteral	IIa	No	Yes
4	Female	3.1	39	Interruption	2	Yes	Parenteral	IIa	No	No
5	Male	2.7	38	HLHS	4	No	Enteral	IIb	Yes	Yes
6	Female	2.9	39	Ebstein	12	No	Enteral	IIa	No	No
7	Male	2.2	37	HLHS	4	No	Parenteral	IIIb	Yes	Yes
8	Female	2.4	37	HLHS	8	No	Parenteral	IIb	No	Yes
9	Female	3.2	40	Truncus arteriosus	4	Yes	Enteral	IIa	No	No
10	Female	2.8	37	VSD-PA	3	Yes	Enteral	IIIa	Yes	No
11	Male	2.9	39	PA/IVS	5	No	Parenteral	IIa	No	No
12	Male	2.45	37	TGA	2	Yes	Enteral	IIb	No	Yes
13	Male	2.3	37	TGA	3	No	Enteral	IIa	No	No
14	Female	2.6	38	VSD-PA	4	Yes	Enteral	IIa	No	No
15	Male	2.7	38	Interruption	5	No	Enteral	IIa	No	No
16	Female	3	40	HLHS	2	Yes	Parenteral	IIa	No	Yes
17	Female	2.9	39	RAI	6	No	Enteral	IIb	Yes	Yes
18	Male	2.8	38	Tricuspid atresia	3	Yes	Parenteral	IIa	No	Yes

DILV: Double inlet left ventricle, HLHS: Hypoplastic left heart syndrome, PA: pulmonary atresia, PA/IVS: Pulmonary Atresia with Intact Ventricular Septum, RAI: Right atrial isomerism, TGA: Transposition of the great arteries, VSD: Ventricular septal defect.

**Table 3:** Antibiotic Exposure Before and After Necrotizing Enterocolitis (NEC), Blood Culture Results, and Duration of Antimicrobial Therapy.

Pt	CRP at NEC Diagnosis (mg/L)	Blood Culture	Antibiotics Before NEC	Antibiotics After NEC
1	18	Negative	None	Ampicillin/sulbactam + Cefotaxime (7 d)
2	24	Negative	None	Ampicillin/sulbactam + Cefotaxime (10 d)
3	20	Negative	None	Ampicillin/sulbactam + Cefotaxime (7 d)
4	36	Negative	Cefazolin prophylaxis (24 h)	Ampicillin/sulbactam + Cefotaxime (7 d)
5	78	*Klebsiella pneumoniae*	Cefazolin prophylaxis (24 h)	Ampicillin/sulbactam + Cefotaxime (2 d) Meropenem + Amikacin (14 d)
6	15	Negative	None	Ampicillin/sulbactam + Cefotaxime (7 d)
7	115	*Klebsiella pneumoniae*	Cefazolin prophylaxis (24 h)	Ampicillin/sulbactam + Cefotaxime (1 d) Meropenem + Amikacin (14 d)
8	40	Negative	Ampicillin/sulbactam + Cefotaxime (5 d)	Ampicillin/sulbactam + Cefotaxime (10 d)
9	24	Negative	Cefazolin prophylaxis (24 h)	Ampicillin/sulbactam + Cefotaxime (7 d)
10	85	*Enterococcus faecalis*	Cefazolin prophylaxis (24 h)	Ampicillin/sulbactam + Cefotaxime (2 d) Ampicillin + Gentamicin (14 d)
11	24	Negative	None	Ampicillin/sulbactam + Cefotaxime (7 d)
12	32	*Staphylococcus epidermidis*	Ampicillin/sulbactam + Cefotaxime (4 d)	Vancomycin + Cefotaxime+ Amikacin (10 d)
13	18	Negative	None	Ampicillin/sulbactam + Cefotaxime (7 d)
14	8	Negative	Cefazolin prophylaxis (24 h)	Ampicillin/sulbactam + Cefotaxime (7 d)
15	54	*Klebsiella pneumoniae*	Ampicillin/sulbactam + Cefotaxime (6 d)	Meropenem+ Amikacin (10 d)
16	50	Negative	Cefazolin prophylaxis (24 h)	Ampicillin/sulbactam + Cefotaxime (10 d)
17	72	*Candida albicans*	Ampicillin/sulbactam + Cefotaxime (7 d)	Fluconazole + Ampicillin/sulbactam + Cefotaxime (14 d)
18	20	*Negative*	Cefazolin prophylaxis (24 h)	Vancomycin + Cefotaxime (7 d)

**Table 4:** Independent Predictors of Necrotizing Enterocolitis in Term Neonates with Critical Congenital Heart Disease.

Variables	*p*	Adjusted Odds Ratio	95% Confidence Interval
Birth weight < 2.5 kg	**0.021**	4.1	1.2–13.4
Gestational age < 38 weeks	**0.018**	3.9	1.3–12.0
Mechanical ventilation dependency	**0.004**	5.1	1.7–15.2
Single ventricle	**0.007**	5.8	1.6–20.7

## Data Availability

The data presented in this study are available from the corresponding author upon reasonable request.

## References

[1] Hoffman J.I., Kaplan S. (2002). The incidence of congenital heart disease. J. Am. Coll. Cardiol..

[2] Khalil M., Jux C., Rueblinger L., Behrje J., Esmaeili A., Schranz D. (2019). Acute therapy of newborns with critical congenital heart disease. Transl. Pediatr..

[3] Choi G.-J., Song J., Kim H., Huh J., Kang I.-S., Chang Y.S., Sung S.I., Hyun M.C. (2022). Development of necrotizing enterocolitis in full-term infants with duct dependent congenital heart disease. BMC Pediatr..

[4] Marino B.S., Tabbutt S., MacLaren G., Hazinski M.F., Adatia I., Atkins D.L., Checchia P.A., DeCaen A., Fink E.L., Hoffman G.M. (2018). Cardiopulmonary resuscitation in infants and children with cardiac disease: A scientific statement from the American Heart Association. Circulation.

[5] Spinner J.A., Morris S.A., Nandi D., Costarino A.T., Marino B.S., Rossano J.W., Shamszad P. (2020). Necrotizing enterocolitis and associated mortality in neonates with congenital heart disease: A multi-institutional study. Pediatr. Crit. Care Med..

[6] McElhinney D.B., Hedrick H.L., Bush D.M., Pereira G.R., Stafford P.W., Gaynor J.W., Spray T.L., Wernovsky G. (2000). Necrotizing enterocolitis in neonates with congenital heart disease: Risk factors and outcomes. Pediatrics.

[7] Gong X., Chen X., Wang L., Zhang M., Nappi F., Zampi J.D., Zheng J., Xu Z., Bao N. (2022). Analysis of clinical features of neonates with congenital heart disease who develop necrotizing enterocolitis: A retrospective case-control study. Ann. Transl. Med..

[8] Tan Recep B.Z., Öztürk E. (2025). Development of necrotizing enterocolitis in term newborns with critical congenital heart disease and affecting factors. Pediatr. Neonatol..

[9] Cognata A., Kataria-Hale J., Griffiths P., Maskatia S., Rios D., O’Donnell A., Roddy D.J., Mehollin-Ray A., Hagan J., Placencia J. (2019). Human milk use in the preoperative period is associated with a lower risk for necrotizing enterocolitis in neonates with complex congenital heart disease. J. Pediatr..

[10] Lau P.E., Cruz S.M., Ocampo E.C., Nuthakki S., Style C.C., Lee T.C., Wesson D.E., Olutoye O.O. (2018). Necrotizing enterocolitis in patients with congenital heart disease: A single center experience. J. Pediatr. Surg..

[11] del Castillo S.L., McCulley M.E., Khemani R.G., Jeffries H.E., Thomas D.W., Peregrine J., Wells W.J., Starnes V.A., Moromisato D.Y. (2010). Reducing the incidence of necrotizing enterocolitis in neonates with hypoplastic left heart syndrome with the introduction of an enteral feed protocol. Pediatr. Crit. Care Med..

[12] Hillyer M., Fundora M., Williams F., Gleason M., Lukacs M., Hamrick S., Meisel J., Clarke S., Korcinsky-Tillman N., Chanani N. (2025). Standardizing the diagnosis of necrotizing enterocolitis in infants with congenital heart disease. J. Perinatol..

[13] Iannucci G.J., Oster M.E., Mahle W.T. (2013). Necrotising enterocolitis in infants with congenital heart disease: The role of enteral feeds. Cardiol. Young.

[14] Jenkins K.J., Gauvreau K., Newburger J.W., Spray T.L., Moller J.H., Iezzoni L.I. (2002). Consensus-based method for risk adjustment for surgery for congenital heart disease. J. Thorac. Cardiovasc. Surg..

[15] Niño D.F., Sodhi C.P., Hackam D.J. (2016). Necrotizing enterocolitis: New insights into pathogenesis and mechanisms. Nat. Rev. Gastroenterol. Hepatol..

[16] Neu J., Walker W.A. (2011). Necrotizing enterocolitis. N. Engl. J. Med..

[17] Asztalos I.B., Hill S.N., Nash D.B., Schachtner S.K., Palm K.J. (2025). Cardiogenic necrotizing enterocolitis in infants with congenital heart disease: A systematic review and meta-analysis. Pediatr. Cardiol..

[18] Moschino L., Guiducci S., Duci M., Meggiolaro L., Nardo D., Bonadies L., Salvadori S., Verlato G., Baraldi E. (2024). Noninvasive tools to predict necrotizing enterocolitis in infants with congenital heart diseases: A narrative review. Children.

[19] Becker K.C., Hornik C.P., Cotten C.M., Clark R.H., Hill K.D., Lenfestey R.W., Smith P.B. (2015). Necrotizing enterocolitis in infants with ductal-dependent congenital heart disease. Am. J. Perinatol..

[20] Bubberman J., van Zoonen A., Bruggink J., van der Heide M., Berger R., Bos A., Kooi E., Hulscher J. (2019). Necrotizing enterocolitis associated with congenital heart disease: A different entity?. J. Pediatr. Surg..

[21] Kelleher S.T., Coleman J., McMahon C.J., James A. (2024). Outcomes and characteristics in term infants with necrotising enterocolitis and CHD. Cardiol. Young.

[22] Maayan-Metzger A., Itzchak A., Mazkereth R., Kuint J. (2004). Necrotizing enterocolitis in full-term infants: Case–control study and review of the literature. J. Perinatol..

[23] Bolisetty S., Lui K., Oei J., Wojtulewicz J. (2000). A regional study of underlying congenital diseases in term neonates with necrotizing enterocolitis. Acta Paediatr..

[24] Baxi A.C., Josephson C.D., Iannucci G.J., Mahle W.T. (2014). Necrotizing enterocolitis in infants with congenital heart disease: The role of red blood cell transfusions. Pediatr. Cardiol..

[25] Balati K., Xu Z., Zhu L., Gong X. (2025). Clinical characterization of necrotizing enterocolitis in neonates with or without congenital heart disease: A case–control study. Ital. J. Pediatr..

[26] Carlo W.F., Kimball T.R., Michelfelder E.C., Border W.L. (2007). Persistent diastolic flow reversal in abdominal aortic Doppler-flow profiles is associated with an increased risk of necrotizing enterocolitis in term infants with congenital heart disease. Pediatrics.

[27] Chan B., Woodbury A., Hazelwood L., Singh Y. (2025). Feeding approach to optimizing nutrition in infants with congenital heart disease. J. Cardiovasc. Dev. Dis..

[28] Kashif H., Abuelgasim E., Hussain N., Luyt J., Harky A. (2021). Necrotizing enterocolitis and congenital heart disease. Ann. Pediatr. Cardiol..

[29] Luo L., Liu X., Yu H., Luo M., Jia W., Dong W., Lei X. (2022). Red blood cell transfusions post diagnosis of necrotizing enterocolitis and the deterioration of necrotizing enterocolitis in full-term and near-term infants: A propensity score adjustment retrospective cohort study. BMC Pediatr..

[30] Singh R., Shah B.L., Frantz I.D. (2012). Necrotizing enterocolitis and the role of anemia of prematurity. Semin. Perinatol..

[31] Khashu M., Dame C., Lavoie P.M., De Plaen I.G., Garg P.M., Sampath V., Malhotra A., Caplan M.D., Kumar P., Agrawal P.B. (2022). Current understanding of transfusion-associated necrotizing enterocolitis: Review of clinical and experimental studies and a call for more definitive evidence. Newborn.

[32] Zacharias P., Blinci J., Shenoy R., Lee J., Singh Y. (2025). Impact of the Pre-Operative Standardized Nutritional Protocol in Infants with Congenital Heart Disease (CHD). J. Cardiovasc. Dev. Dis..

[33] Pradhan S., Strohacker C., Schachtner S., Palm K., Trauth A., Gao Z., Marcuccio E. (2024). Management of hematochezia in infants with congenital heart disease admitted to the acute care cardiology unit: A multicenter retrospective pilot study. J. Pediatr..

[34] Murphy C., Baskind S., Aladangady N., Banerjee J. (2023). Measuring gut perfusion and blood flow in neonates using ultrasound Doppler of the superior mesenteric artery: A narrative review. Front. Pediatr..

[35] Touloukian R.J., Posch J.N., Spencer R. (1972). The pathogenesis of ischemic gastroenterocolitis of the neonate: Selective gut mucosal ischemia in asphyxiated neonatal piglets. J. Pediatr. Surg..

[36] Roychaudhuri S., Grewal G., Vijayashankar S.S., Lavoie P., Maheshwari A. (2022). Necrotizing enterocolitis associated with congenital heart disease—A review article. Newborn.

